# Opportunities to engage in positive activities during the COVID-19 pandemic: perspectives of individuals with mood disorders

**DOI:** 10.1016/j.jad.2023.10.135

**Published:** 2023-10-27

**Authors:** Katherine Gordon-Smith, Chloe Hampshire, Berenice Mahoney, Amy Perry, Katie J.S Lewis, Nick Craddock, Ian Jones, Lisa Jones

**Affiliations:** 1 Psychological Medicine, University of Worcester, UK; 2 School of Psychology, University of Worcester, UK; 3 National Centre for Mental Health, Division of Psychological Medicine and Clinical Neurosciences, Cardiff University, UK; 4 Department of Psychiatry, Brigham & Women’s Hospital, Harvard Medical School, Boston, MA USA

**Keywords:** Bipolar disorder, Major Depressive Disorder, Covid-19 pandemic, Patient perspective, Self-management.

## Abstract

**Background:**

Despite cross-sectional population and clinical studies finding individuals with existing mood disorders being adversely impacted by the COVID-19 pandemic, longitudinal studies have not shown a worsening of psychiatric symptoms. In response to these findings, we explored opportunities to engage in positive activities during the pandemic from the perspectives of individuals with mood disorders.

**Methods:**

A bespoke survey, containing closed and open questions, was sent to participants with mood disorders who were part of the UK Bipolar Disorder Research Network (BDRN). Questions related to experiences of positive impacts of the pandemic, levels of engagement in positive activities and coping strategies.

**Results:**

Response rate was 46.4% (N=1688). 61.9% reported positive life changes during the pandemic, with slower pace of life reported most frequently (52.8%). 47.3% reported no adverse impact of the pandemic on implementing their usual coping strategies. Activities that respondents most commonly reported the same or greater level of engagement in compared to before the pandemic were avoiding known mood triggers (82.3%), relaxation techniques (78.8%) and the ability to maintain set routines (69.4%).

**Limitations:**

Responder bias may be present and experiences during the pandemic are likely to differ among other clinical and research mood disorders cohorts.

**Conclusions:**

Our findings may help to explain why longitudinal studies have not found a worsening of mental health symptoms during the COVID-19 pandemic. Identifying potential facilitators to maintaining mental health have wider applicability, and may help to inform future evidence-based psychoeducation and self-management programmes for mood disorders.

## 1 Introduction

At the outset of the coronavirus disease-19 (COVID-19) pandemic in 2020, identification of pandemic-related outcomes among individuals living with serious mental illness was designated a public health priority because the pre-existence of a psychiatric disorder was expected to increase the risk of impaired functioning and adverse psychological experiences (Holmes et al., 2020; Hotopf et al., 2020). The effects of pandemic-related restrictions on biological and social rhythms raised specific concerns for individuals living with mood disorders with predictions of an exacerbation of psychiatric symptoms (Rajkumar, 2020; Stefana et al., 2020) and increased risk of relapse (Rajkumar, 2020). Cross-sectional population studies that have followed have suggested that people with existing mental illness have been more psychologically impacted by the pandemic, reporting higher levels of depression, anxiety and stress, than those with no psychiatric history (Alonzi et al., 2020; Asmundson et al., 2020; Kwong et al., 2021; Pierce et al., 2021; Sanchez et al., 2023). Similarly, a cross-sectional study in the early stages of the pandemic which focused on individuals with psychotic disorders and bipolar disorder (BD) found the majority of participants experienced low levels of wellbeing and high levels of mental health difficulties (Barrett et al., 2022).

Interestingly, however, findings from the smaller number of longitudinal studies among individuals with existing mental illness, including those with mood disorders, have shown no significant worsening of mood symptoms during the pandemic compared to pre-pandemic symptoms (Czysz et al., 2021; Leightley et al., 2021; Lewis et al., 2022; Pinkham et al., 2020; Yocum et al., 2021). For example, in an early study among 148 individuals with severe mental illness (92 with schizophrenia spectrum illnesses and 56 with mood disorders) mood symptoms and sleep were stable after five months from the start of the pandemic (Pinkham et al., 2020). Similarly, more recently, we found that among 356 individuals with BD providing weekly ratings using an online mood monitoring tool, symptoms of depression, mania, insomnia and suicidal thoughts did not significantly differ on average during the pandemic in 2020 compared to pre-pandemic in 2019 (Lewis et al., 2022). A further recent study in individuals with BD found an increase in (hypo)manic symptoms in the initial phase of the pandemic which then decreased (Koenders et al., 2021).

There are several possible explanations for longitudinal studies not finding a worsening of symptoms among samples of individuals with mood disorders during the pandemic. For example, the lifestyle constraints imposed during parts of the COVID-19 pandemic might stabilise mood symptoms in some individuals with an existing mood disorder by removing triggers. Furthermore, it is possible that enforced reduced social contact during the pandemic had less impact on some individuals with mood disorders, whose symptoms already caused them to limit their social contacts. Another possible explanation is that the findings might reflect well-established self-management and coping strategies among particular cohorts of individuals living with mood disorders. Previous small scale qualitative studies of individuals with pre-existing mental health conditions, including those with BD and major depressive disorder (MDD), reported that some participants described a positive impact on their mental health as a result of the pandemic (Burton et al., 2021; Gillard et al., 2021). For example, some participants reported that the reduced pace of life helped with mental health symptoms and allowed more time for activities (Gillard et al., 2021). However, to date, opportunities to engage in activities and life changes that could have a positive impact on mental health, from the perspectives of individuals with mood disorders have not been explored in a large sample - which was the aim of the current study. Better understanding will not only contribute to mental health and coping advice provided to individuals with mood disorders in future situations where socially-restrictive protective public health measures are required, but will also contribute more generally to evidence-based psychoeducation about living and coping with mood disorders.

## 2 Method

### 2.1 Participants

Participants were part of the UK Bipolar Disorder Research Network (BDRN). BDRN is an ongoing research programme into the genetic and environmental aetiology of BD and related mood disorders. Participants are recruited throughout the UK via the National Health Service (NHS) and via advertisements through patient support organisations, such as Bipolar UK (www.bipolaruk.org.uk). Inclusion criteria are that participants are: (i) aged 18 years or over at participation; (ii) able to provide written informed consent; (iii) meet Diagnostic and Statistical Manual of Mental Disorders-IV (DSM-IV) criteria (American Psychiatric Association, 2000) for a mood disorder; and (iv) onset of mood symptoms before the age of 65 years. Best-estimate main lifetime diagnosis is made according to DSM-IV criteria based on an in-depth interview using the Schedules for Clinical Assessment in Neuropsychiatry (Wing et al., 1990) and review of psychiatric and general practice (primary care) case-notes where available. The research has approval from the West Midlands NHS Research Ethics Committee (MREC/97/7/01) and all participating NHS Trusts and Health Boards. Written informed consent is obtained from all participants.

### 2.2 COVID-19 survey

A bespoke online and paper survey was sent to BDRN participants in current contact with the research programme (N= 3639) in May 2021. The survey contained Likert response questions relating to: i) whether the COVID-19 pandemic had led to any positive life changes that benefitted mental health; ii) experiences of implementing usual coping strategies during the pandemic; and, iii) level of engagement in positive activities during the pandemic compared to before the pandemic with follow-up optional open text boxes. Questions in the survey were based on current COVID-19 literature at the time of designing the questionnaire, our research to date within the field of mood disorders and input from a research champion with a lived experience of bipolar disorder. The survey questions, which were all in relation to the timeframe of since the start of the COVID-19 pandemic, were developed by the authors iteratively to ensure a range of potential experiences and perspectives were included with the survey being piloted by our research champion in the final stages of development.

### 2.3 Data analysis

Descriptive statistics were used to describe the sample and responses to the quantitative survey items. Quantitative survey responses were further compared between those with BD and MDD. Demographic variables were compared between survey responders and non-responders. Non-parametric statistical tests were used (chi-square tests for categorical data and Mann-Whitney *U* tests for continuous data) as data were not normally distributed. Examples were identified from the open-text boxes to enhance the quantitative survey responses.

## 3 Results

### 3.1 Sample

Of the 3639 individuals invited to complete the survey, 1688 (46.4%) responded. The sample of 1688 individuals is described in Table 1. 71.3% were female with a median age of 59 years. The majority, 88.2%, had a DSM-IV diagnosis of BD: bipolar I disorder (54.9%), bipolar II disorder (27.5%), schizoaffective disorder bipolar type (2.5%), bipolar disorder not otherwise specified (3.3%). 11.8% had recurrent MDD. At the time of completion of the survey 31.7% respondents reported that they were living alone and 31.7% reported having caring responsibilities for family members. 37.4% respondents were employed or self-employed prior to COVID-19 restrictions, 32.7% were retired, 18.5% were unable to work and 11.4% reported ‘other’ (which included being a student and volunteering). Individuals who completed the survey were significantly older (59 vs. 54 years, p=<0.001) than those who did not, and a significantly higher proportion of responders compared to non-responders were female (71.3% vs 68.2%, p=0.043).

### 3.2 Positive life changes during the pandemic

Overall, 61.9% of the sample reported that the pandemic had led to positive changes in their life that benefitted mental health (46.8% reporting ‘a few’ and 15.1% reporting ‘a lot’) compared to 31.1% reporting the pandemic had not led to any positive changes. Individuals with BD were significantly more likely to report positive benefits compared to those with MDD (62.8% vs 55.1%, p=0.036). Table 2 summarises the positive changes that participants endorsed from a predetermined list with the option of selecting all that applied. The most commonly indicated positive change was a slower pace of life (52.8%), followed by more time at home (43.7%), having more time to reflect (41.3%), spending more time outdoors (33.9%), reconnecting with friends and family (21.7%), and more flexible working (15.6%). Examples of additional comments made by individuals are given in Table 2. Compared to those with MDD, a significantly higher proportion of individuals with BD reported the following as a positive change: i) more time to reflect (42.4% vs 32.7%, p=0.008); ii) more time at home (44.6% vs 37.2%, p=0.048); and, iii) reconnecting with friends and family (22.6% vs 14.6%, p=0.010).

### 3.3 Implementation of usual coping strategies during the COVID-19 pandemic

47.7% of respondents reported that they had found implementing their usual coping strategies easier or no harder during the pandemic and 52.3% reported that it was harder (Table 3). There were no significant differences according to diagnosis (BD compared to MDD). Facilitators of implementing coping strategies reported by participants included having social support/networks, mood monitoring, and the strength of pre-pandemic self-management routines (Table 3).

### 3.4 Level of engagement in positive activities during the pandemic compared to pre-pandemic

Table 4 summarises level of engagement in positive activities respondents reported during the COVID-19 pandemic in comparison to pre-pandemic. The activities are listed in descending order of the percentage of respondents reporting more or the same level of engagement during the pandemic: avoiding known mood triggers (82.3%), relaxation techniques (78.8%), having a set routine (69.4%), getting enough sleep (67.7%), spending time outside (63.5%), physical exercise (60.5%), volunteering (59.1 %), other leisure activities (57.5%) and religious activities (56.7%). Examples are provided in Table 4 where respondents engaging either more/same or less in each activity during the pandemic gave additional details. Comparisons of level of engagement in positive activities according to diagnosis indicated that the only significant difference was a higher proportion of individuals with BD were significantly more likely to report getting more or the same level of enough sleep (vs. less) compared to those with MDD (69.1% vs 57.4%, p=0.001).

## 4 Discussion

This study is the first to explore the perspectives of a large sample of individuals with mood disorders (N=1688) about opportunities to engage in positive activities during the COVID-19 pandemic. Strengths include the size of our well-characterised UK-wide sample, and that our data collection took place later in the pandemic (May – Aug 2021) allowing us to capture experiences that may differ from those in studies conducted earlier in the pandemic.

Overall nearly two thirds (62%) of the sample reported that the pandemic had led to at least some positive changes in their life which benefitted their mental health, with just over half (53%) reporting that a slower pace of life was a positive change and over one third reporting positive benefits of having more time at home (44%), having more time to reflect (41%) and spending more time outdoors (34%). Previous qualitative research into the experiences of 49 individuals with mental health problems, including mood disorders (N=28), reported that some individuals described a positive impact on their mental health during the pandemic which overlapped with our findings and included enjoyment of a quieter world, slowing down and more time for activities and self-care (Gillard et al., 2021). Approximately half of our sample (48%) reported that implementing their usual coping strategies during the pandemic was unaffected or easier than pre-pandemic. The activities that respondents most commonly reported the same or greater level of engagement in compared to before the pandemic were avoiding known mood triggers (82.3%), relaxation techniques (78.8%) and the ability to maintain set routines (69.4%). Some activities were particularly adversely affected by the pandemic for our sample, most notably ability to engage in religious activities, leisure activities, hobbies, volunteering, and physical exercise.

Our findings may partly help to explain previous longitudinal studies that have overall found no significant worsening of mood symptoms among samples of individuals with BD and MDD during the pandemic compared to pre-pandemic (Czysz et al., 2021; Leightley et al., 2021; Lewis et al., 2022; Pinkham et al., 2020; Yocum et al., 2021). However, there was heterogeneity among participants about whether or not the pandemic had led to any positive changes and also variation in their ability to implement usual coping strategies and engage in positive activities during the pandemic. This in particular is highlighted by approximately half of respondents indicating their usual coping strategies had been harder to implement. This heterogeneity is likely to be due to a number of factors, including the facilitators highlighted by our respondents. For example, a number of individuals specifically reported social support as facilitating implementing coping strategies, with one participant explicitly mentioning they felt they would have found it difficult to self-manage their mood without their family. Further post hoc analysis in our sample revealed that a non-significantly higher proportion of those living alone (56%) reported finding usual coping strategies harder to implement compared to those living with others (50%) (data not shown). This is consistent with previous research investigating the social networks of individuals living with BD which has found perceived higher social support to be associated with lower levels of psychological distress (Warren et al., 2018) and enhanced mood management (Owen et al., 2017).

Particularly in BD, but also in mood disorders more widely, disturbances in social and circadian rhythms (patterns in waking, sleeping exercising, socialising) have been associated with the occurrence of mood episodes and symptoms (Levenson et al., 2015; Shen et al., 2008; Szuba et al., 1992). In a recent large (N=997) cross-sectional survey with a self-reported mood disorder diagnosis (MDD or BD), 79.1% of participants indicated that social disruption related to the COVID-19 pandemic affected the regularity of their daily routines at least to some extent, which was associated with increased levels of depressive symptoms (Kahawage et al., 2022). This figure is higher than the third of participants in our sample who reported less ability to maintain a set routine during the pandemic and is likely to be due to the use of different measures (a single item in our survey vs. a questionnaire measure of social rhythms). Data collection from the surveys in the two studies were also at different time points in the pandemic, with the survey in Kahawage et al (2022) taking place relatively early in the pandemic (April-September 2020) and our survey later in May-August 2021. Participants in our sample were also older (59 years on average vs. 40 years) and as a result routines may have been disrupted less, although we found no association between age and reported changes in set routine among our respondents (data not shown). In a further paper by Kahawage et al (2022b) the authors analysed the free-text questions within their survey asking about participants’ subjective experiences of the COVID-19 social changes including in relation to disrupted daily routines. One of the four themes identified was that many participants described the role that social interaction had played in maintaining their routines prior to the COVID-pandemic and the impact of this being altered. This finding may also help to further explain our finding that a number of individuals reported that social support facilitated implementing coping strategies. It is possible that social support may have played a role for some individuals in helping to maintain their daily routines.

Although we did not find many significant differences according to BD v MDD diagnosis, individuals with BD were significantly more likely to report overall positive changes during the pandemic and to report having more time to reflect, more time at home and reconnecting with friends and family as specific positive changes for their mental health compared to those with MDD. A higher proportion of those with BD were also significantly more likely to report getting enough sleep during the pandemic at the same level or more. Sleep and sleep disturbances are known to play a key role in BD (Plante and Winkelman, 2008) and in the triggering of mood episodes particularly (hypo)mania (Lewis et al., 2017). Furthermore, previous qualitative research of self-management strategies reported by individuals with BD found that getting sufficient and regular sleep was one of the most important reported strategies for maintaining or regaining wellness (Gordon-Smith et al., 2021; Suto et al., 2010). It is therefore possible that maintaining sleep routines may form an important part of self-management for a proportion of individuals with BD which may, in part, explain our finding.

### 4.1 Limitations and future directions

Participants were a sub-sample of the larger BDRN cohort. Despite a good survey response rate of 46.4%, those not responding may have had different experiences during the pandemic. The majority of participants were female (71%), had a DSM-IV diagnosis of bipolar I disorder (55 %) and a median age of 59 years which is likely to limit the generalisability of the results. Furthermore, our study was only conducted within the UK and among participants motivated to be part of an established ongoing research programme. Although participants were not receiving any kind of psychoeducational support from BDRN, a proportion of respondents, 37.6%, were enrolled on the BDRN True Colours online mood-monitoring tool for research (Gordon-Smith et al., 2019). Although it was not a focus of the current study to investigate this, it is possible that the use of True Colours as a mood monitoring tool aided self-management during the pandemic for some participants. It is likely that experiences during the pandemic would differ among other clinical and research mood disorders cohorts both within and outside the UK. Furthermore, we did not collect information relating to current mood state at the time of survey completion, receipt of pharmacological or other kinds of therapies during the pandemic, the presence of current comorbidities such as anxiety disorders or substance use/dependence, or family bereavement which may have influenced our findings. In addition, we were unable to accurately know what proportion of respondents had COVID-19 by the time of survey completion due to variations in testing capacity within the UK over the timeframe covered in the survey.

Identifying potential facilitators to maintaining mental health and coping is important to inform psychoeducation and self-management programmes for individuals with mood disorders as the pandemic continues to evolve and beyond. Furthermore, the findings may contribute to advice provided to individuals in other situations where socially-restrictive protective public health measures are required, for example, following natural disasters. A slower pace of life was the most commonly reported positive life change of the pandemic reported by participants suggesting this might be a factor to explore further in relation to mood management programmes. For example, in a recent qualitative study exploring patient’s perspectives of the effects of a group-based therapeutic patient education programme for bipolar disorder, individuals reported slowing down the pace of their lives as a strategy implemented to manage their disorder following the programme (Duval et al., 2022). Furthermore, factors that enabled individuals to engage in the same or increased level of relaxation techniques, avoid known mood triggers and maintain routines would be useful to explore in further detail. For example, it is not clear from our data whether high level of engagement was in response to the challenges of the pandemic, or whether the restrictions associated with the pandemic offered more opportunity to engage in these activities. Among individuals with BD in particular, the impact of the pandemic on sleep and the specific facilitators and barriers to getting enough sleep could help inform future self-management strategies. The heterogeneity in responses also highlights the importance of healthcare professionals being able to identify those who may face more challenges in access to engaging in positive activities and facilitators to coping such as social support.

## Figures and Tables

**Table 1:** Demographic and diagnostic characteristics of individuals completing the Bipolar Disorder Research Network COVID-19 Survey (N=1688)

Sample	N=1688
Female n (%)	1203 (71.3)
Current age, years: median (IQR)	59 (18)
**DSM-IV diagnosis**	
Bipolar I disorder n (%)	927 (54.9)
Bipolar II disorder n (%)	464 (27.5)
Schizoaffective disorder bipolar type n (%)	43 (2.5)
Bipolar disorder not otherwise specified n (%)	55 (3.3)
Major depressive disorder recurrent n (%)	199 (11.8)
**Demographic variables at completion of survey**	
Living alone n (%)	529/1670* (31.7)
Caring responsibilities n (%)	528/1663* (31.7)
**Employment status – prior to COVID-19 restrictions**	
Employed or self-employed n (%)	627/1678* (37.4)
Retired n (%)	548/1678* (32.7)
Unable to work n (%)	311/1678* (18.5)
Other n (%)	192/1678* (11.4)

IQR=interquartile range

* Totals vary due to unknown information.

**Table 2:** Positive life changes during the pandemic reported by participants (N=1688)

	N (%)	Examples of additional comments
***Slower pace of life***	891 (52.8)	*Slower pace of life lifts my mood*. *Slower pace of life helps particularly if high in mood, I can tend to take on too much so allows me to pace myself*.
***More time at home***	738 (43.7)	*Enjoyed being quiet at home reading, watching films and art*. *Guilt-free uninterrupted time at home has allowed me to address major issues such as lifestyle and coping strategies*.
***Having more time to reflect***	697 (41.3)	*I’ve had time to reflect on what does and doesn't work in my life*. *The pandemic has caused me to reflect on what is truly important in life*.
***Spending more time outdoors***	572 (33.9)	*Biggest benefit by far being able to get outdoors during daylight hours in winter. This definitely benefitted my mood*. *I have spent a lot of time in my garden, and growing things, which is on my toolkit of super useful good things to do to nurture my mental wellbeing, so that’s all been nice*.
***Reconnecting with friends and family***	366 (21.7)	*I have reconnected with friends from my life. There is no reason why we should have lost touch, but we have been brought together through a real appreciation for friendship and contact and I am sure this will continue*. *I can socialise through zoom with some friends I previously only saw once a year because of distance between us*.
***More flexible working***	264 (15.6)	*Flexible working practices have had a lasting effect on my mood*. *I have benefitted from not having to commute because it’s quite tiring, it means I can get up later, it means I’m at home in the evening, it means I can use my lunch hour to go for a walk*.

**Table 3:** Implementation of usual coping strategies during the pandemic reported by participants (N=1518*)

	N (%)
**Usual coping strategies easier or no harder to implement**	724 (47.7)
**Usual coping strategies harder to implement**	794 (52.3)
**Facilitators of implementing coping strategies reported**	
**Social support/networks**	*My self-management is reasonably good…I think that would have been a different story if I hadn’t had the family around me*.
	*I think I’m quite lucky in that I’ve got existing really good support networks in place*.
	*I think definitely having somebody…it’s something about being part of each other’s every day. And supporting each other, sort of the reciprocal support that happens*.
	*My wife’s support, really, and my son. She’s been great. She has really looked after me*.
**Mood monitoring**	*My mood monitoring tool is useful in that aspect because it’s enabled me to track what’s going on, on a weekly basis because I can look back to think, “well I got a low score that day, why did I get a low score?” That’s the kind of foundations, the kind of structure around managing mood I think helps*.
**Strength of pre-pandemic self-management routines**	*So I kind of worked on stuff that I really, really strongly had in place because of courses around, you know, how to manage…and I also teach mental health first aid, so I’m quite well versed in things to do to help*.
	*I’d say it’s from pre-pandemic, which is my toolkit of all the things I do to help myself feel good...I’ve kind of built them all into my routine anyway by now, which is great. But particularly when I am challenged, or I feel my mood’s under threat, then I’ll really remind myself*.

* Total varies from the 1688 completing the survey as there was an unsure/not applicable option.

**Table 4:** Level of engagement in positive activities during the pandemic compared to pre-pandemic reported by participants

	More or the same engagement	Example	Less engagement	Example
	N (%)		N (%)	
Avoiding known mood triggers	1215/1477 (82.3)	*Because of lockdown I have avoided stressful situations, easier to reduce anxiety*.	262/1477 (17.7)	*I have discovered that using more social media is a potent trigger for mental distress and mood changes*.
Relaxation techniques (for example, mediation or yoga)	672/853 (78.8)	*Did a lot of online pilates classes which I really enjoyed*.	181/853 (21.2)	*Everything stopped when lockdown began. I used to attend meditation classes, used to practice yoga*.
Having a set daily routine	1016/1464 (69.4)	*Being allowed to impose a set daily routine has transformed my life*.	448/1464 (30.6)	*It has totally messed with my routines and left me at a complete loose end*.
Getting enough sleep	1106/1633 (67.7)	*I sleep more so I feel better*.	527/1633 (32.3)	*I got fixated on alternative media so I had very poor sleep and high mood*.
Spending time outside	1016/1600 (63.5)	*Nature and being in the fresh air were important to me prior to the pandemic, but I have become even more aware how that helps me to deal with mental health*.	584/1600 (36.5)	*Barely been anywhere or done anything outside of the home since pandemic*.
Physical exercise	913/1510 (60.5)	*Increased physical activity including running walking and gardening. Probably contributed to stable mental health*.	597/1510 (39.5)	*I normally swim to help with my mental health but can’t do that now*.
Volunteering/ helping others	564/955 (59.1)	*I have started volunteering and this has given my life more focus and structure, which has been very beneficial for me*.	391/955 (40.9)	*Had to stop volunteering at my local charity shop*.
Other leisure activities/hobbies	828/1439 (57.5)	*Played bridge online which has been very beneficial to my mental health*.	611/1439 (42.5)	*Fighting to get through each day when home-schooling. So no free time or time for myself*.
Religious activities	333/587 (56.7)	*Before the pandemic I was unable to pursue religious activities due to poor physical health. Now during pandemic I can access these online at home*.	254/587 (43.3)	*Being an active member of a choir and a church, the loss of these activities has been source of regret*.

* Totals vary as not applicable in all cases for example if individual did not engage in activity before the pandemic.

## Highlights

- Opportunities to engage in positive activities during the pandemic assessed among 1688 individuals with mood disorders.
- 62% reported positive life changes during the pandemic, including slower pace of life.
- Approximately half reported usual coping strategies unaffected or facilitated by pandemic.
- Majority reported no adverse impact of the pandemic on level of engagement in positive activities.
- Reduced opportunities to engage in positive activities during the pandemic reported by some respondents.
